# Facile Preparation of Rod-like MnO Nanomixtures via Hydrothermal Approach and Highly Efficient Removal of Methylene Blue for Wastewater Treatment

**DOI:** 10.3390/nano9010010

**Published:** 2018-12-22

**Authors:** Yuelong Xu, Bin Ren, Ran Wang, Lihui Zhang, Tifeng Jiao, Zhenfa Liu

**Affiliations:** 1 State Key Laboratory of Metastable Materials Science and Technology, Yanshan University, Qinhuangdao 066004, China; xudalong.cool@163.com; 2 Hebei Key Laboratory of Applied Chemistry, School of Environmental and Chemical Engineering, Yanshan University, Qinhuangdao 066004, China; wr1422520780@163.com; 3 Institute of Energy Resources, Hebei Academy of Sciences, Shijiazhuang 050081, China; RENBINTS@126.com (B.R.); zlhkxy@126.com (L.Z.); 4 Hebei Engineering Research Center for Water Saving in Industry, Shijiazhuang 050081, China

**Keywords:** hydrothermal method, manganese oxide, adsorption, degradation, nanomixtures

## Abstract

In the present study, nanoscale rod-shaped manganese oxide (MnO) mixtures were successfully prepared from graphitic carbon nitride (C_3_N_4_) and potassium permanganate (KMnO_4_) through a hydrothermal method. The as-prepared MnO nanomixtures exhibited high activity in the adsorption and degradation of methylene blue (MB). The as-synthesized products were characterized by scanning electron microscopy (SEM), transmission electron microscopy (TEM), surface area analysis, X-ray diffraction (XRD), and X-ray photoelectron spectroscopy (XPS). Furthermore, the effects of the dose of MnO nanomixtures, pH of the solution, initial concentration of MB, and the temperature of MB removal in dye adsorption and degradation experiments was investigated. The degradation mechanism of MB upon treatment with MnO nanomixtures and H_2_O_2_ was studied and discussed. The results showed that a maximum adsorption capacity of 154 mg g^−1^ was obtained for a 60 mg L^−1^ MB solution at pH 9.0 and 25 °C, and the highest MB degradation ratio reached 99.8% under the following optimum conditions: 50 mL of MB solution (20 mg L^−1^) at room temperature and pH ≈ 8.0 with 7 mg of C, N-doped MnO and 0.5 mL of H_2_O_2_.

## 1. Introduction

Water pollution is currently among the major environmental challenges and has attracted increasing research attention. The wide use of dyes has resulted in organic pollution in water, and dyes are considered a severe threat to ecosystems [1,2,3,4,5,6]. As untreated dyes are very active and stable, adsorption followed by oxidative degradation has emerged as a practical and effective technique to accelerate the treatment of dye effluent pollution. Thus, the following technological systems have been developed for the removal of dyes from water: physical adsorption [7], biodegradation [8,9] and chemical reaction and adsorption [10]. In recent years, photocatalytic decomposition [11,12,13] and chemical oxidation reduction have become highly efficient techniques for the degradation of methylene blue (MB) in water.

Over the last decades, nanomixtures, mostly nanorods/nanotubes-like structured, have been widely used for contaminant adsorption/removal [14,15,16,17]. Cavallaro et al. [15] investigated comprehensively the effect of anionic surfactants (sodium dodecanoate and sodium dodecylsulfate) on pristine halloysite nanotubes (HNT), which was beneficial for the solubilization and delivery of hydrophobic compounds from such hybrid materials. Recently, the oxidation degradation of dyes in water using environmentally benign oxidants has attracted considerable attention [18,19,20,21]. On this basis, some nontoxic and low-cost metal oxides have been widely used as catalysts for the oxidation of organic compounds [22,23,24,25]. Huang et al. [26] reported the application of Prussian blue (PB)-modified γ-Fe_2_O_3_ magnetic nanoparticles (PBMNPs) in the degradation of MB. The PBMNPs were used as peroxidase-like catalysts with H_2_O_2_ as the oxidant to completely degrade MB. The optimal conditions were as follows: pH range of 3 to 10, degradation temperature of 25 °C and degradation time of 120 min. However, the preparation process for the PBMNPs was very complicated and involved the use of toxic chemicals. Wolski et al. [27] investigated the effects of ZnO, Nb_2_O_5_ and ZnNb_2_O_6_ on the degradation of dyes, and MB could be completely degraded under optimal conditions. Nevertheless, the as-reported metal oxides (Nb_2_O_5_ and ZnNb_2_O_6_) were highly toxic and expensive.

In recent years, the synergistic application of metal oxides and H_2_O_2_ as peroxidase-like catalysts and an oxidant, respectively, in the degradation of dyes has been reported. Metal oxides can catalyze the generation of active oxygen (such as hydroxyl radicals (HO^•^), peroxides (HO_2_^-^) and superoxide anions (HO_2_^•^)) upon H_2_O_2_ treatment, and this active oxygen can catalyze the degradation of dyes in water [28]. Saha et al. reported a novel method to prepare nanodimensional copper ferrite which exhibited high activity in the degradation of dyes in water with H_2_O_2_ as an oxidant [29]. The researchers used ethylenediaminetetraacetic acid and citric acid as the complexing agent and the fuel, respectively, in a modified complexometric method to prepare CuFe_2_O_4_, which had the capability to degrade 96% of the total MB. Because of its size-, structure- and morphology-dependent characteristics, and the variety of unique physical, chemical and functional properties, hausmannite (MnO) has been widely investigated in the fields of materials science, chemistry and physics. Zhang et al. prepared MnO nanocrystals of various sizes and shapes by soft-template self-assembly and studied the synthetic conditions and degradation mechanism of MB with H_2_O_2_ treatment [30]. In their report, cetyltrimethylammonium bromide (CTAB), polyvinyl pyrrolidine (PVP) and P123 were used as structure-directing agents; manganese sulfate was used as the source of manganese; and the size and shape of MnO could be controlled by varying the growth time, reaction temperature, surfactant, and manganese source. The as-prepared MnO showed a very high capacity for (above 99.7%) MB degradation.

Recently, Because of its excellent chemical and thermal stabilities and nontoxicity, graphitic carbon nitride (g-C_3_N_4_) [31,32,33,34,35], a novel 2D material, which was prepared through simple and green pyrolysis of melamine, has been used in many applications, such as energy conversion, biomedical applications and hydrogen production. According to the literature, g-C_3_N_4_ can absorb aromatic pollutants via the conjugated π region, which makes g-C_3_N_4_ a potential effective adsorbent. In this paper, the preparation of MnO Nanomixtures through a hydrothermal method with C_3_N_4_ as the source of carbon and nitrogen and potassium permanganate (KMnO_4_) as the source of manganese was investigated. The effects of the hydrothermal reaction time, molar ratio of C_3_N_4_ to KMnO_4_, and hydrothermal temperature on the adsorption capacity for MB were studied. In addition, the adsorption and degradation properties of the as-prepared product were systematically studied, and thermodynamic and kinetic analyses of the adsorption–degradation process were performed through experiments.

## 2. Materials and Methods

### 2.1. Materials

All reagents were purchased from Shanghai Aladdin Bio-Chem Technology Co., Ltd, Shanghai, China. All reagents were of analytical reagent (AR) grade and were used as received without further treatment.

### 2.2. Synthesis of C_3_N_4_

C_3_N_4_ was prepared by heating melamine (10 g) at 650 °C for 4 h in an air atmosphere. After the heat treatment, a light yellow solid was obtained.

### 2.3. Synthesis of MnO Nanomixtures

MnO nanomixtures were prepared via a hydrothermal method with C_3_N_4_ as the source of carbon and nitrogen and potassium permanganate (KMnO_4_) as the source of manganese. Typically, certain amounts of C_3_N_4_ power and KMnO_4_ were put into a 100 mL hydrothermal reactor. The molar ratios of C_3_N_4_ to KMnO_4_ were 2.0, 4.0 and 6.0, and the mass concentration of the reactants (C_3_N_4_ + KMnO_4_) in the solution was 12%. The hydrothermal temperature was set as 180 °C, and the hydrothermal reaction times were 24 h and 30 h. The as-prepared MnO nanomixtures with a hydrothermal reaction time of 30 h were denoted MnO-X (X = 2, 4, and 6), where X represents the reactants molar ratio of C_3_N_4_ to KMnO_4_. The sample prepared with a molar ratio of 4.0 and a hydrothermal reaction time of 24 h was denoted as MnO-24.

### 2.4. MB Adsorption and Degradation Experiments

In the adsorption experiments, 50 mL of 10−60 mg L^−1^ MB aqueous solutions containing 5 mg of the MnO nanomixtures adsorbent were stirred at different temperatures (293.15−333.15 K) and different pH values (3.0–11.0) for MB adsorption. After an adsorption time of 20–300 min, the adsorbent solution was centrifuged, and the supernatant was examined by a UV-Vis spectrophotometer (TU-1900, Beijing Persee Instruments Co. Ltd., Beijing, China) to determine the MB concentration. The maximum wavelength of MB absorption was observed at λ = 665 nm.

The reusability of the MnO nanomixtures adsorbent was also investigated via 10 consecutive adsorption/desorption cycles. Briefly, the MnO nanomixtures with MB adsorbed were stirred in 50 mL of HCl solution (0.1 M) for 120 min, and then, the adsorbent was washed three times with distilled water. The adsorbed MB was desorbed from the MnO nanomixtures adsorbent, and the recovered MnO nanomixtures adsorbent was used to adsorb MB in another cycle. This cycle of adsorption and desorption was performed 10 times. The amount of MB adsorbed (q_t_) was calculated according to Equation (1):

(1) $$q_{t}=\frac{\left(C_{0}-C_{t}\right)V}{W}$$

where C_0_ is the initial concentration of MB (mg L^−1^), C_t_ is the concentration of MB at contact time t (mg L^−1^), V is the volume of the MB solution (L), and W is the weight of the adsorbent (g).

The MB degradation process was carried out in a 100 mL beaker containing 50 mL of a MB dye solution (20 mg L^−1^ or 40 mg L^−1^), 0.5 mL of 30% H_2_O_2_, and 7 mg of MnO nanomixtures. The degradation time was varied from 0 h to 24 h, and the MB concentration was monitored by a UV-Vis spectrophotometer.

### 2.5. Characterization

MnO nanomixtures were characterized by X-ray diffraction (XRD, SMART LAB, Rigaku, Akishima, Japan) with CuKa radiation (λ = 1.54 Å), scanning electron microscopy (SEM, Field Emission Gun FEI QUANTA FEG 250, FEI Corporate, Hillsboro, OR, USA), transmission electron microscopy (TEM, HT7700, High-Technologies Corp., Ibaraki, Japan) and X-ray photoelectron spectroscopy (XPS, ESCALAB 250Xi XPS, Thermo Fisher Scientific, San Jose, CA, USA). The Brunauer−Emmett−Teller (BET) method was utilized to calculate the specific surface areas (ASAP2420 surface area analyzer, Micromeritics, Norcross, GA, USA). The pore volume and pore size were calculated from the adsorption–desorption isotherms using the Barrett−Joyner−Halenda (BJH) model. The total pore volume (V_total_) was estimated from the amount adsorbed at a relative pressure (P/P_0_) of 0.998.

### 2.6. Kinetic, Adsorption and Degradation Isotherm Models

The kinetics of the adsorption process were studied through kinetic models in our work. The pseudo-first-order kinetic model (2) and pseudo-second-order kinetic model (3) were adopted to fit the experimental data.

(2) $$\ln\left(q_{e}-q_{t}\right)={\mathrm{lnq}}_{e}-k_{1}t$$

(3) $$\frac{t}{q_{t}}=\frac{1}{k_{2}{q_{e}}^{2}}+\frac{t}{q_{e}}$$

In these equations, q_e_ represents the equilibrium absorption capacity (mg g^−^^1^), q_t_ represents the absorption amount (mg g^−^^1^) at an absorption time of t (min), and k_1_ and k_2_ are the pseudo-first-order rate constant (min^−^^1^) and the pseudo-second-order rate constant (g mg^−1^·min^−1^), respectively.

The Langmuir isotherm model (4) was adopted to investigate the surface properties, adsorbate affinity and adsorption capacity of MnO nanomixtures.

(4) $$\frac{C_{e}}{q_{e}}=\frac{1}{q_{m}b}+\frac{C_{e}}{q_{m}}$$

In this equation, q_e_ (mg g^−1^) is the equilibrium adsorption capacity, q_m_ (mg g^−1^) is the maximum adsorption capacity (corresponding to complete monolayer coverage), C_e_ (mg L^−1^) is the adsorbate concentration at the adsorption equilibrium, and b (L mg^−1^) is a constant. The kinetics of the degradation process was also investigated via the pseudo-first-order kinetic model (2).

### 2.7. Thermodynamic Evaluation of the Adsorption Process

The thermodynamics of the adsorption process were obtained from Equations (5)–(7).

(5) $$K_{c}=\frac{q_{e}}{C_{e}}$$

(6) $$\Delta G^{0}=-{\mathrm{RTlnK}}_{c}$$

(7) $${\mathrm{lnK}}_{c}=\frac{\Delta S^{0}}{R}-\frac{\Delta H^{0}}{\mathrm{RT}}$$

In these equations, ΔG^0^ is the standard Gibbs free energy change, ΔH^0^ is the standard enthalpy change, ΔS^0^ is the standard entropy change, q_e_ is the equilibrium adsorption capacity, C_e_ (mg L^−1^) is the adsorbate concentration at the adsorption equilibrium, Kc is the distribution coefficient, R is the molar gas constant (8.314 J mol^−1^ K^−1^), and T is the adsorption temperature (K).

## 3. Results and Discussion

**Figure 1:**
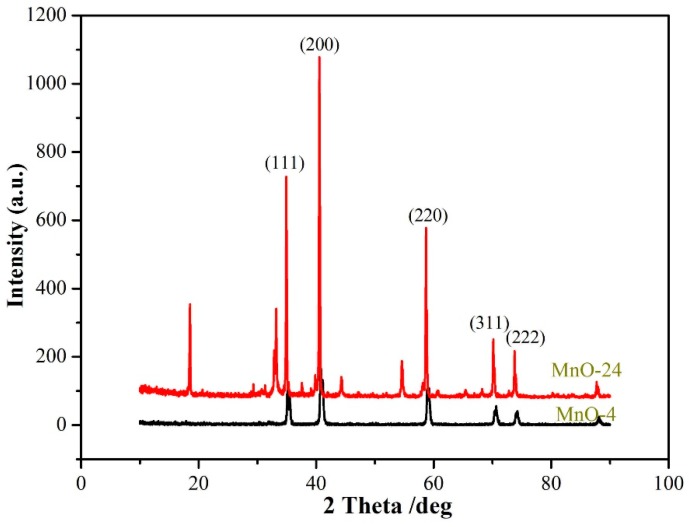
XRD patterns of the as-prepared MnO nanomixtures samples.

The XRD patterns of the as-prepared MnO nanomixtures samples are shown in Figure 1. As presented in Figure 1, the peaks of (111), (200), (220), (311) and (222) were attributed to MnO [36], which indicated that MnO nanomixtures were successfully prepared via a novel hydrothermal self-assembly method. We also investigated the effect of the hydrothermal reaction time on the formation of MnO nanomixtures. We found that other manganese oxides were produced when the hydrothermal reaction time was less than 30 h. In the experiment, manganese oxide was the only product when the hydrothermal reaction time exceeded 30 h.

**Figure 2:**
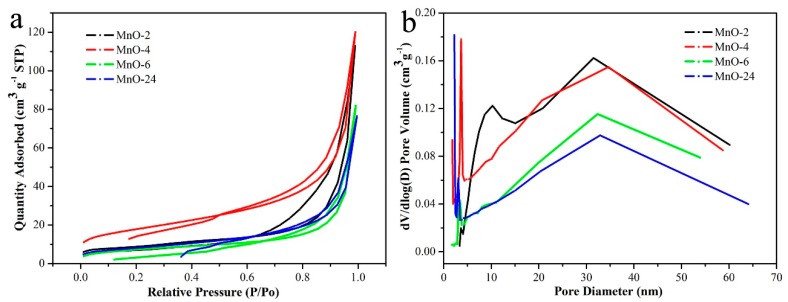
Nitrogen adsorption–desorption isotherms (**a**) and pore size distributions (**b**).

The nitrogen adsorption–desorption isotherms are shown in Figure 2a, and the pore size distribution curves are shown in Figure 2b. As seen in Figure 2a, all the curves corresponded to type-IV isotherms, and hysteresis loops could be clearly observed, illustrating the presence of a pore structure. The high P/P_0_ of the hysteresis loops indicated a large pore size distribution, which was in accordance with the pore size distribution curves. As shown in Figure 2b, the as-prepared MnO nanomixtures samples exhibited a micro-mesoporous structure. The surface properties, consisting of the specific surface area (S_BET_), micropore surface area (S_micro_), average pore diameter (D_average_) and total pore volume (V_total_), are listed in Table 1. MnO-4 showed the largest surface area and total pore volume, which were beneficial for adsorption. As presented in Table 1, the molar ratio of C_3_N_4_ to KMnO_4_ and the hydrothermal reaction time exerted obvious effects on the textural properties, in which shorter hydrothermal reaction times and higher or lower molar ratios affected the hydrothermal self-assembly process.

**Figure 3:**
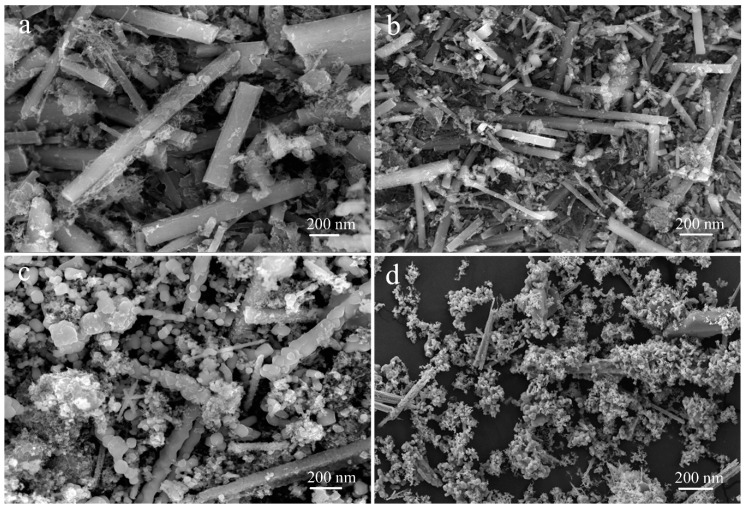
Images of MnO-2 (**a**), MnO-4 (**b**), MnO-6 (**c**) and MnO-24 (**d**).

**Figure 4:**
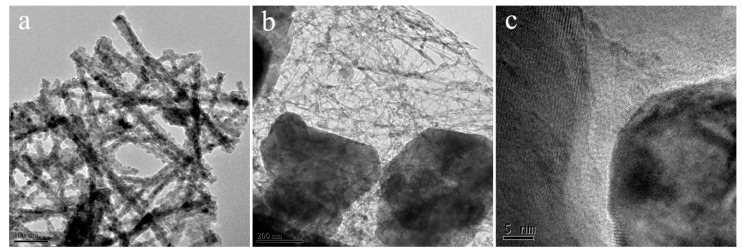
TEM images (**a**,**b**) and high resolution image (**c**) of MnO-4.

SEM images of the as-prepared MnO nanomixtures samples and TEM images of MnO-4 are shown in Figure 3 and Figure 4. The nanoscale rod-shape of C, N-doped MnO can be clearly seen in Figure 3; this product was formed via the polymerization of C_3_N_4_ and oxidation by KMnO_4_. As shown in Figure 3d, the amount of rod-shaped MnO nanomixtures particles in MnO-24 was less than that in the other samples, which was caused by the shorter hydrothermal reaction time. When the molar ratio of C_3_N_4_ to KMnO_4_ was more than 4.0, many linked spherical particles were formed, as shown in Figure 3c; there particles formed through the polymerization of excess C_3_N_4_ in the hydrothermal process. As presented in Figure 4a,b, nanoscale rod-shaped MnO nanomixtures particles were clearly observed. The lattice fringe spacing was determined from Figure 4c and was attributed to the presence of manganese.

**Figure 5:**
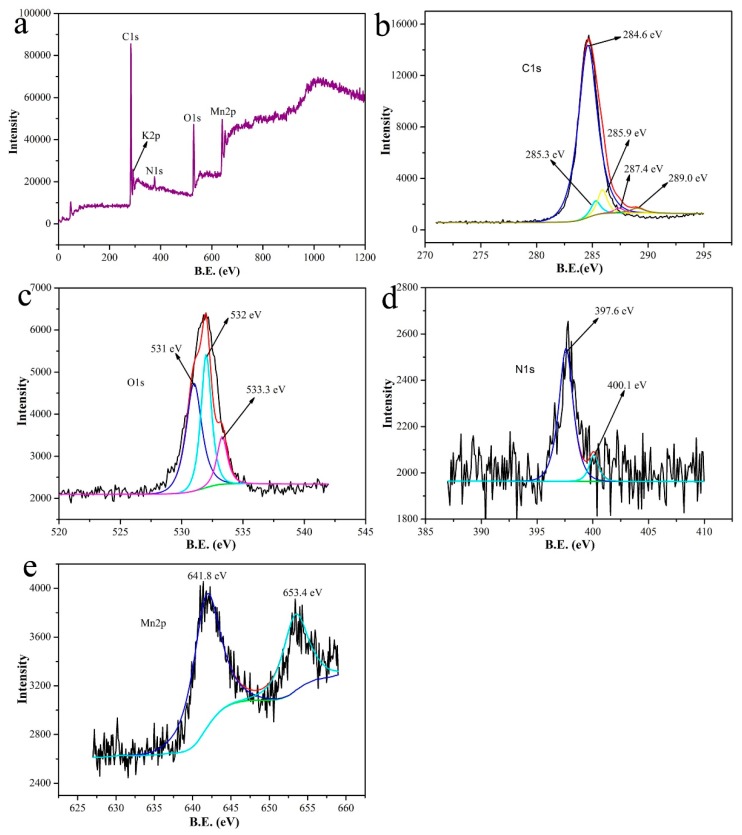
XPS spectra of MnO-4: (**a**) full-scan spectrum, (**b**) C 1s spectrum, (**c**) O 1s spectrum, (**d**) N 1s spectrum, and (**e**) Mn 2p spectrum.

XPS was performed to analyze the chemical nature of MnO-4; the results are shown in Figure 5. Figure 5a reveals the presence of C, K, O, N and Mn, which corresponded to peaks at 285 eV, 300 eV, 535 eV, 410 eV and 650 eV, respectively. As presented in Figure 5b, five peaks were observed (284.6 eV, 285.3 eV, 285.9 eV, 287.4 eV and 289.0 eV), and these peaks were attributed to C–N–C, C–C, C–O, C=O, and O–C=O groups. This result indicated that C_3_N_4_ was oxidized by KMnO_4_ in the hydrothermal self-assembly process. The peaks shown in Figure 5c corresponded to C=O (531.0 eV), COOH (532.0 eV) and C–O–C (535.0 eV). As shown in Figure 5d, two peaks [7] were observed at 400.3 eV and 398.8 eV, which were assigned to N–C_3_ and C–N–C, respectively. The presence of N–C_3_ was beneficial for MB adsorption [37]. The peaks at 641.8 eV and 653.4 eV corresponded to Mn 2p, which indicated the presence of manganese.

**Figure 6:**
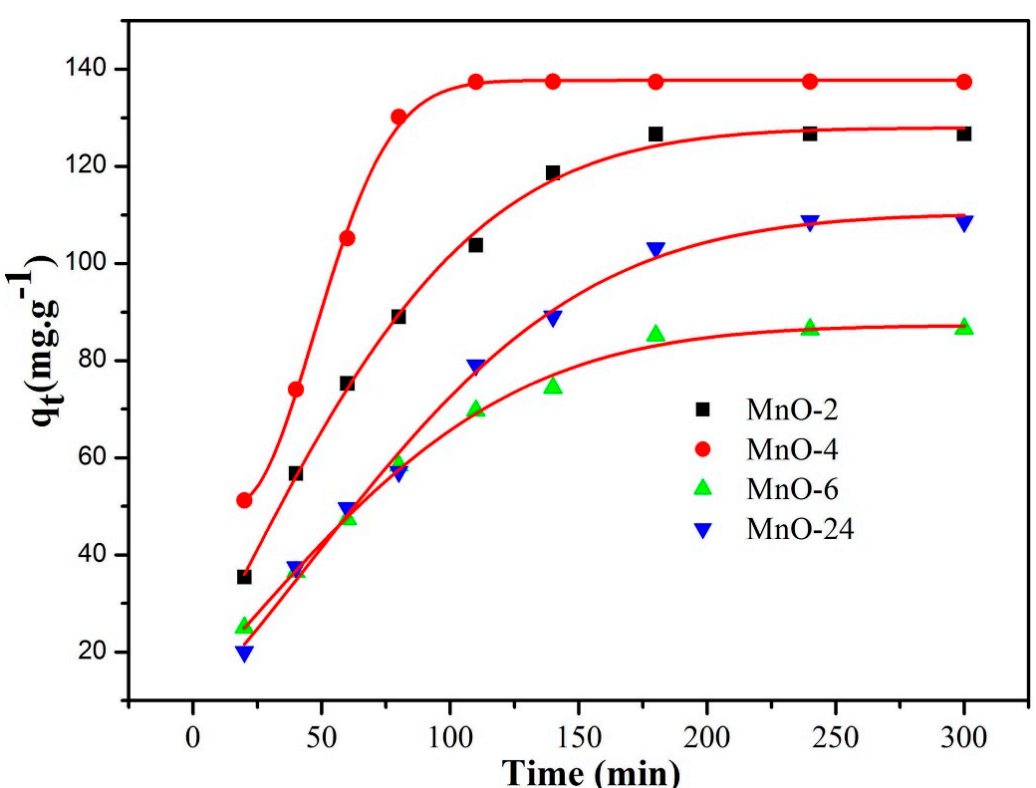
MB adsorption curves of the as-prepared samples.

The effect of the different samples on the MB adsorption amount was investigated, and the result is shown in Figure 6. As seen in Figure 6, MnO-4 and MnO-6 exhibited larger adsorption amounts than MnO-2, which was attributed to the higher reactant molar ratio of C_3_N_4_ to KMnO_4_. C_3_N_4_ introduced a π-conjugation system in MnO nanomixtures during the hydrothermal process, which could improve the adsorption capacity. Meanwhile, a moderate dosage of KMnO_4_ could improve the surface area to increase the adsorption of MB. From the comparison of the adsorption amounts of MnO-4 and MnO-24, the hydrothermal reaction time exerted an effect on the adsorption capacity, in which MnO-4 had a higher adsorption capacity of up to 137 mg g^−1^ in a 20 mg L^−1^ MB solution at 20 °C. The zeta potentials of MnO-2, MnO-4, MnO-6 and MnO-24 in water were as follows: −29.8 mV, −42.3 mV, −37.1 mV, and −34.7 mV, respectively. MB is a cationic dye; thus, the lower the zeta potential is, the better the adsorption. 

**Figure 7:**
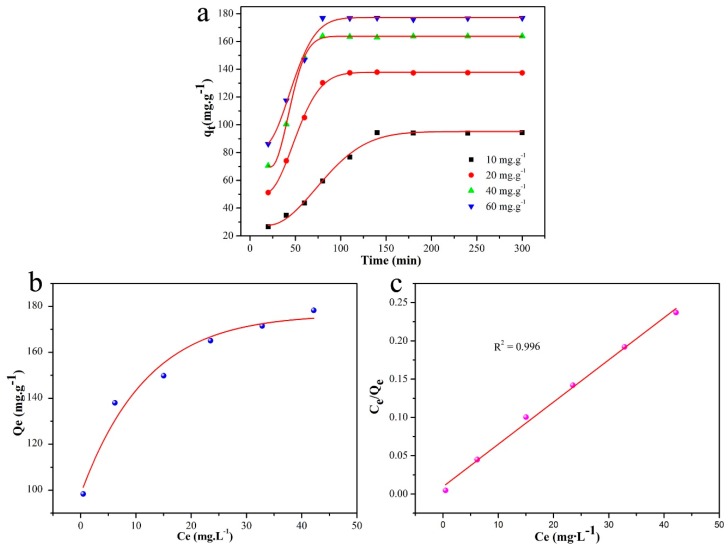
(**a**) Adsorption curves under different concentrations of MB; (**b**) adsorption isotherm of an MB solution in MnO-4; (**c**) Langmuir isotherm plot for MB adsorption in MnO-4.

The effect of the MB concentration on the adsorption capacity is shown in Figure 7a, in which the adsorption capacity was observed to increase with the MB concentration. The higher the MB concentration, the shorter the adsorption equilibrium time was. The MB adsorption efficiency was up to 96% for an MB concentration of 10 mg L^−1^ at 150 min. As seen in Figure 7b, an equilibrium plateau was reached, which indicated that MnO-4 acted as a monolayer adsorbent in MB absorption. The Langmuir model was adopted to investigate the adsorption process on the MnO-4 surface, and the results are shown in Figure 7c. The correlation coefficient (R^2^) of the fitted curve was 0.996, which indicated that adsorption occurred through a Langmuir process, meaning that it was a monolayer process. This analysis result was in accordance with the results of Figure 7b.

**Figure 8:**
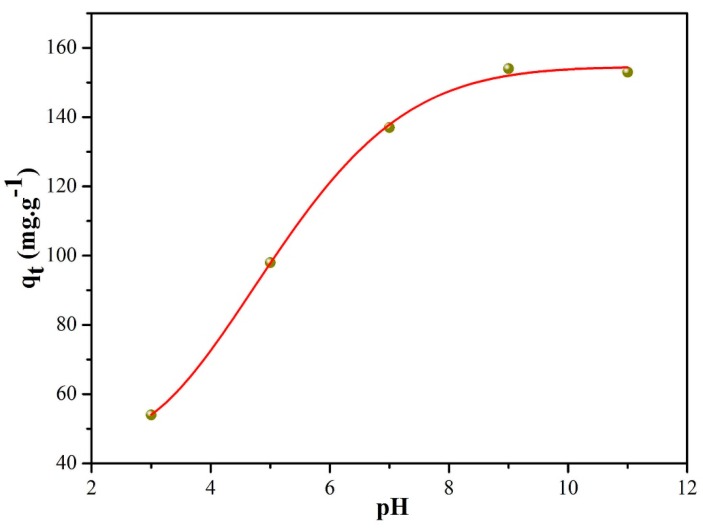
MB adsorption capacity of MnO-4 at different pH values.

The effect of the MB solution pH on the adsorption capacity was studied, and the results are presented in Figure 8, in which the maximum adsorption capacity was achieved with a strong basic MB solution and the adsorption capacity increased with the solution pH. This result was attributed to the electrostatic interaction between the MB molecules and MnO nanomixtures. In the previous discussion, the zeta potentials exerted an effect on the adsorption capacity, as MB is a cationic dye. In an acidic solution, the zeta potentials of MnO nanomixtures were positive, which inhibited MB adsorption. In contrast, at lower pH values, the zeta potentials were negative and lower. Therefore, MnO-4 had a high adsorption capacity in a basic MB solution. Meanwhile, the nitrogen doping of MnO could improve the alkalinity of the solution, which was beneficial for MB adsorption.

**Figure 9:**
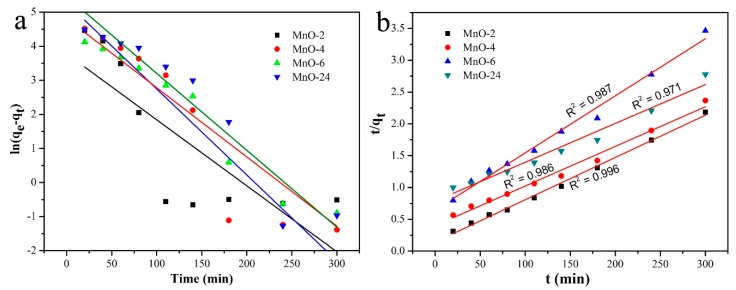
Pseudo-first-order kinetic model plot (**a**) and pseudo-second-order kinetic model plot (**b**).

The pseudo-first-order and pseudo-second-order kinetic models were used to analyze the kinetics of the adsorption process. The theoretical adsorption capacity of MnO-4 calculated from the pseudo-first-order model was 194 mg g^−1^, and that calculated from the pseudo-second-order model was 164 mg g^−1^ (Table 2), which fit well with the experimental data (154 mg g^−1^). As shown in Figure 9, the R^2^ values obtained from the pseudo-second-order model were better than the R^2^ values obtained from the pseudo-first-order model. In conclusion, the pseudo-second-order model was more suitable for investigation of the MB adsorption process.

**Figure 10:**
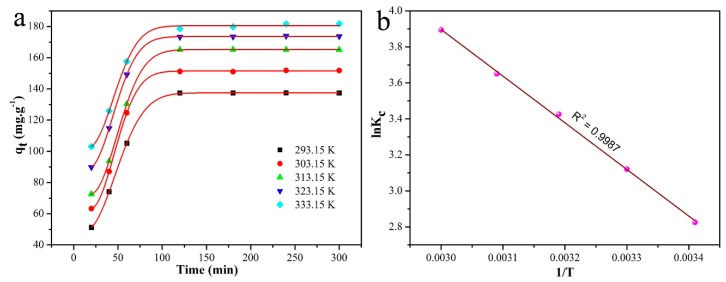
Adsorption curves measured at different temperatures (**a**) and the plot of ln Kc versus 1/T for MnO-4 (**b**).

MB adsorption experiments were performed at different temperatures, and the results are shown in Figure 10a. At the same time, the plot of ln Kc versus 1/T for MnO-4 is demonstrated in Figure 10b. As presented in Figure 10a, a higher adsorption capacity was obtained at a higher temperature, which indicated that a high temperature was beneficial for MB adsorption. The ∆G^0^, ∆H^0^ and ∆S^0^ values of MB adsorption on MnO-4 were calculated from Equations (6) and (7) [38] to be −7.4 kJ mol^−1^, 21.5 kJ mol^−1^ and 97.0 J mol^−1^, respectively. The value of ∆G^0^ was negative, which demonstrated that spontaneous MB adsorption occurred on the MnO-4 surface. In addition, the value of ∆S^0^ was positive, which was attributed to an increase in the chaos at the adsorbent/solution interface during MB adsorption in MnO-4. In addition, the value of ∆H^0^ was below 40 kJ mol^−1^, as demonstrated by the physisorption of MB in MnO-4, and the positive value indicated that the process was endothermic, which was in accordance with the experimental results.

**Figure 11:**
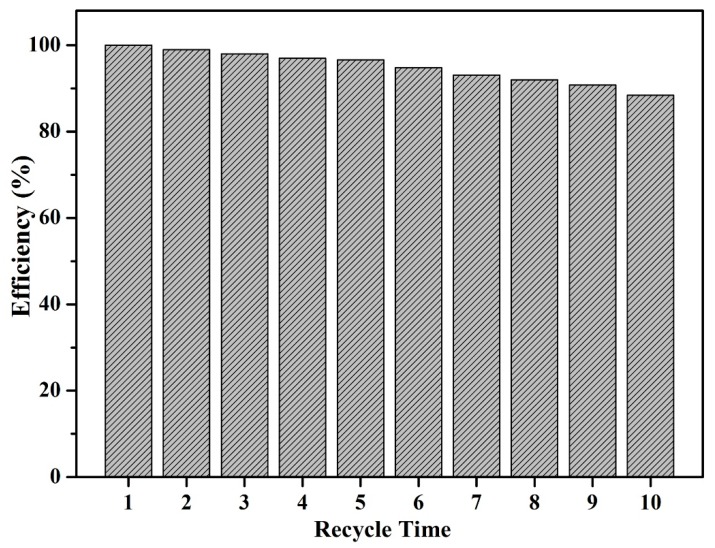
MB adsorption capacities of MnO-4 in 10 adsorption cycles.

Repeated experiments were conducted to investigate the reusability of MnO-4 for MB adsorption, and the results are shown in Figure 11. The adsorption capacity was 137 mg g^−1^ in the first cycle, and 96% of the adsorption capacity, corresponding to 132 mg g^−1^, was retained in the last cycle. Therefore, this reusability indicated that MnO-4 was a good adsorbent for MB. Meanwhile, the obtained MnO-4 exhibited excellent adsorption capacity, which could be roughly compared with other reported absorbents shown in Table 3.

**Figure 12:**
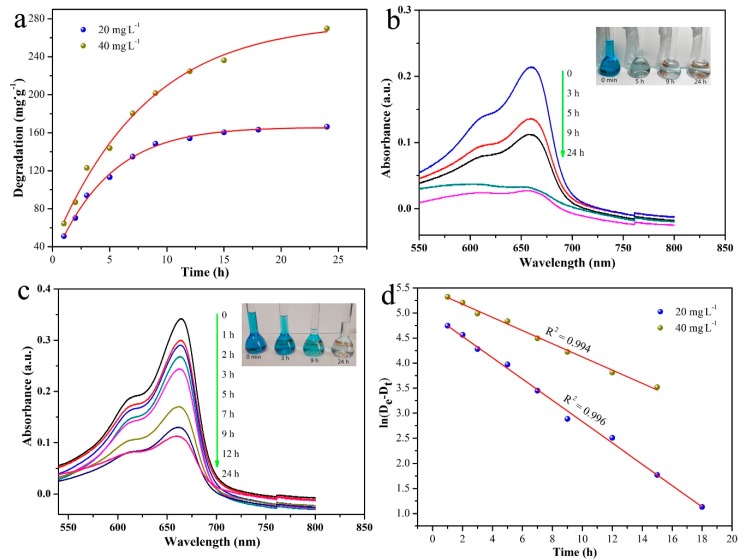
MB degradation curves under different MB concentrations (**a**); UV-Vis spectra of 20 mg L^−1^ MB after various degradation times (**b**); UV-Vis spectra of 40 mg L^−1^ MB after various degradation times (**c**); and pseudo-first-order kinetic model plot of the degradation process (**d**).

The degradation efficiency of MB in MnO nanomixtures was investigated in this work. As shown in Figure 12a, MnO nanomixtures exhibited high degradation efficiency under different MB concentrations (99.8%, ≈142 mg g^−1^ at a MB concentration of 20 mg L^−1^). As presented in Figure 12b,c, the MB solution exhibited a sharp absorption band at 656 nm in the UV-Vis spectrum, and this absorption band obviously decreased with increasing degradation time. The degradation kinetics were well fitted by the pseudo-first-order model shown in Figure 12d, and the theoretical De (the degradation amount at the degradation equilibrium) value was 146 mg g^−1^, which was in good agreement with the experimental data. This analysis result indicated that the pseudo-first-order model could effectively describe the MB degradation process in MnO nanomixtures [45,46,47,48,49,50,51,52].

**Figure 13:**
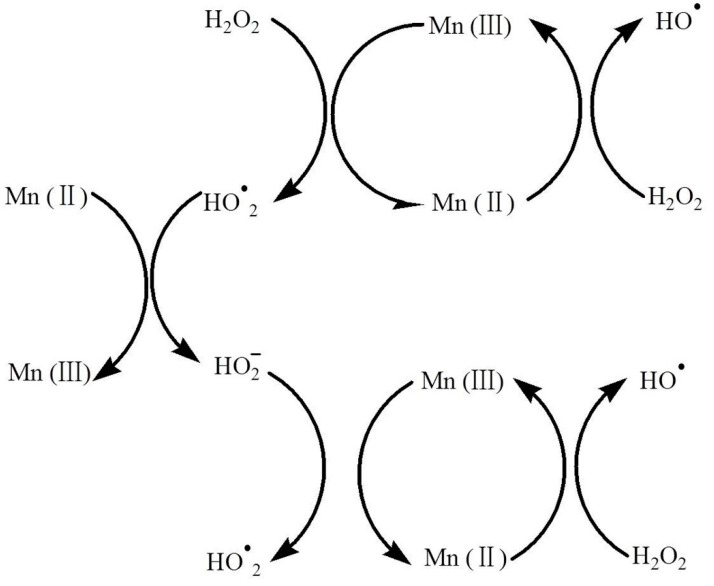
Degradation mechanism of MB in MnO nanomixtures.

The degradation mechanism of MB in MnO nanomixtures was proposed (Figure 13). Active superoxide anions and/or peroxide species could form in the H_2_O_2_-MnO system according to previous reports [16,53], and these species could oxidize MB. As shown in Figure 13, H_2_O_2_ was used as an oxidant to form various superoxide anions and peroxide species, and C; N-doped MnO was used as a catalyst to catalyze the decomposition of H_2_O_2_. Mn(III)/Mn(II) played an important role in the MB degradation process and contributed to ideal MB degradation in C, N-doped MnO. Present obtained MnO nanomixtures demonstrated potential applications in self-assembled materials design and composites for wide applications [54,55,56,57,58,59,60,61,62,63,64,65].

## 4. Conclusions

In summary, novel nanoscale rod-shaped MnO nanomixtures were successfully prepared via a hydrothermal self-assembly method with C_3_N_4_ as the source of carbon and nitrogen and potassium permanganate (KMnO_4_) as the source of manganese. The as-prepared materials exhibited good MB adsorption and degradation with H_2_O_2_ as the oxidant. The maximum adsorption capacity was 154 mg g^−1^, and the optimum degradation efficiency was 99.8%. The adsorption process was very well fitted by the pseudo-second-order model, and the degradation process was very well fitted by the pseudo-first-order model. MB adsorption occurred through physicorption, and MB degradation was caused by a chemical reaction. Meanwhile, MnO nanomixtures exhibited excellent reusability. The as-prepared MnO nanomixtures are potential and effective materials for extensive pollutant removal.

## Figures and Tables

**Table 1:** Surface characterization of different samples.

Entry	S_BET_ (m^2^/g)	S_micro_ (m^2^/g)	Daverage (nm)	V_total_ (cm^3^/g)
MnO-2	30.6	6.3	22.81	0.175
MnO-4	38.7	8.5	23.09	0.193
MnO-6	35.5	1.0	21.46	0.127
MnO-24	33.9	1.6	15.97	0.168

**Table 2:** Parameters of pseudo-first-order kinetic model and pseudo-second-order kinetic model for the adsorption of MB in MnO nanomixtures.

Entry	Pseudo-First-Order Kinetic Model		Pseudo-Second-Order Kinetic Model	
	K_1_	q_e_ (mg g^−1^)	K_2_	q_e_ (mg g^−1^)
MnO-2	0.019	43.70	0.00031	150.38
MnO-4	0.025	194.03	0.000097	164.12
MnO-6	0.021	122.85	0.00012	111.48
MnO-24	0.022	234.40	0.000047	160.67

**Table 3:** Comparison of the adsorption capacities of different absorbents from previous reports with that of C, N-MnO-4.

Adsorbent	mg g^−1^	Reference
Wheat shells	21.5	[39]
Chitosan-modified zeolite	37	[40]
Fe_3_O_4_@Ag/SiO_2_ nanospheres	128.5	[41]
α-Fe_2_O_3_@carboxyl-functionalized yeast composite	49.5	[42]
N, O-codoped porous carbon	100.2	[43]
Kaolin	52.7	[44]
C, N-doped MnO	154	Present work
